# The effects of intravenous immunoglobulin on intestinal inflammation: a systematic review of animal and clinical studies

**DOI:** 10.7717/peerj.21024

**Published:** 2026-03-25

**Authors:** Tiancheng Chu, Xin Yuan, Tong Wang, Wei Zhang, Xiaochen Yan, Changqing Li, Peng Jiang, Shengliang Ye, Li Ma, Pan Sun, Xi Du, Ping Fu, Zongkui Wang

**Affiliations:** 1 Institute of Blood Transfusion, Chinese Academy of Medical Sciences & Peking Union Medical College, Chengdu, Sichuan, China; 2 Chengdu Rongsheng Pharmaceuticals Co., Ltd, Chengdu, Sichuan, China; 3 Center for Natural Products Research, Chengdu Institute of Biology, Chinese Academy of Sciences, Chengdu, Sichuan, China; 4 Beijing Tiantan Biological Products Co., Ltd., Beijing, China

**Keywords:** Intravenous immunoglobulin, Intestinal inflammation, Inflammatory bowel disease, Radiation enteritis, Chemotherapy-induced mucositis, *Clostridioides difficile*, Animal models, Refractory IBD

## Abstract

**Background:**

Intestinal inflammation encompasses a range of conditions, including inflammatory bowel disease (IBD), radiation-induced enteritis, and chemotherapy- or toxin-induced epithelial injury. Although intravenous immunoglobulin (IVIg) is widely used in autoimmune and inflammatory disorders, its role in these forms of intestinal injury has not been systematically evaluated.

**Objective:**

This systematic review aimed to summarize the existing animal and clinical evidence on the therapeutic effects of IVIg in intestinal inflammation.

**Methods:**

This systematic review was conducted in accordance with the Preferred Reporting Items for Systematic Reviews and Meta-Analyses (PRISMA) 2020 guidelines. Four electronic databases (PubMed, Web of Science, Embase, and China National Knowledge Infrastructure) were systematically searched for studies published from January 1, 2000 to February 3, 2026. Eligible studies included animal and clinical investigations evaluating the role of IVIg in the context of intestinal inflammation. Data were narratively synthesized, and risk of bias was assessed using the SYstematic Review Centre for Laboratory animal Experimentation (SYRCLE) tool for animal studies and Risk Of Bias In Non-randomized Studies - of Interventions (ROBINS-I) for non-randomized clinical studies.

**Results:**

Ten studies were included: seven animal and three clinical studies. IVIg generally exhibited anti-inflammatory and epithelial-protective effects across models of Dextran Sulfate Sodium (DSS)-induced colitis, radiation-induced enteritis, chemotherapy-induced mucositis, and toxin-induced epithelial damage. Reported mechanisms included interleukin (IL)-10 signaling, Fcγ receptor modulation, ferroptosis inhibition, and microbiota-mediated immune regulation. Human data were restricted to small, retrospective, uncontrolled cohorts of patients with refractory or steroid-resistant IBD, often with contraindications to standard immunosuppressive therapy. In these studies, some patients experienced short-term clinical improvement, but responses were variable, long-term outcomes were frequently unsatisfactory, and the overall risk of bias was serious to critical.

**Conclusion:**

Current animal and clinical data suggest that IVIg can modulate intestinal inflammation and epithelial injury but are insufficient to support its use as a standard therapy for IBD or other intestinal inflammatory conditions. At present, IVIg should be considered, at most, as an adjunctive or rescue option in carefully selected, refractory cases in which guideline-recommended therapies are ineffective or contraindicated. Further work is needed to improve the rigour and transparency of preclinical studies and to conduct small, well-designed prospective clinical studies in clearly defined niche indications to clarify any potential role of IVIg within the expanding therapeutic armamentarium for intestinal inflammation. PROSPERO ID (CRD420251051592).

## Introduction

Intestinal inflammation is a hallmark of various gastrointestinal disorders, encompassing both chronic immune-mediated diseases and acute injury-induced conditions. Although the upstream triggers differ substantially, ranging from dysregulated immunity in inflammatory bowel disease (IBD) to cytotoxic injury caused by radiation, chemotherapy, or bacterial toxins, these conditions converge on common downstream pathological events such as disruption of the mucosal barrier, infiltration of immune cells, epithelial cell death, oxidative stress, the release of pro-inflammatory cytokines, and alterations in host-microbiota interactions (Alam & Madan, 2024; Cytlak et al., 2022; Dahlgren & Lennernäs, 2023; Hu et al., 2023; Tang et al., 2025), ultimately compromising intestinal homeostasis and nutrient absorption (Lin et al., 2024). A better understanding of therapeutic agents that modulate these shared inflammatory pathways may therefore provide broader mechanistic insight into mucosal protection.

Among chronic intestinal inflammation, inflammatory bowel disease, comprising Crohn’s disease (CD) and ulcerative colitis (UC), represents a major clinical concern (Tie et al., 2023). It is characterized by relapsing-remitting inflammation, immune dysregulation and tissue remodeling (Lee & Chang, 2021). Conventional treatments for IBD typically include aminosalicylates, corticosteroids, immunosuppressants (*e.g.*, azathioprine, methotrexate), and biologic therapies targeting tumour necrosis factor (TNF) or interleukin pathways (Cai, Wang & Li, 2021; Moschen, Tilg & Raine, 2019). While these therapies have improved disease management, a substantial proportion of patients experience inadequate response, drug intolerance, or loss of efficacy over time (Kim et al., 2021; Raine et al., 2021). These limitations have prompted ongoing efforts for alternative or adjunctive treatments, especially for refractory cases.

Chemotherapy- and radiotherapy-associated intestinal injuries represent major gastrointestinal complications in patients undergoing cancer treatment. A wide range of chemotherapy agents, including anthracyclines, platinum compounds and fluoropyrimidines, lack selectivity for malignant cells and damage rapidly proliferating intestinal epithelial cells (Delgado, Grabinger & Brunner, 2016; Sougiannis et al., 2021). As a result, chemotherapy-induced mucositis frequently manifests as abdominal pain, diarrhea, gastrointestinal bleeding and impaired nutrient absorption (Heindryckx & Sjoblom, 2024), affecting up to one-third of treated patients and often necessitating dose reduction or treatment interruption (Kwon, 2016). Similarly, approximately half of all cancer patients require abdominal or pelvic radiotherapy (Hauer-Jensen, Denham & Andreyev, 2014), placing the radiosensitive intestinal mucosa at substantial risk for radiation-induced enteritis (Gandle, Dhingra & Agarwal, 2020). This condition may present with diarrhea, abdominal pain, or even obstruction, and can significantly compromise the continuation and effectiveness of anticancer therapy (Larrey & Pathak, 2022). Given the clinical burden and the lack of effective therapies for these treatment-related intestinal injuries (Dahlgren et al., 2021; Yang et al., 2023), experimental models of radiation enteritis and chemotherapy-induced mucositis provide important complementary systems for evaluating potential interventions.

Toxin-induced epithelial injury is another important cause of acute intestinal inflammation, most prominently represented by *Clostridioides difficile* (*C. difficile*) infection (Bobo et al., 2013; Guh et al., 2020), a major cause of healthcare-associated infectious colitis. *C. difficile*-associated colitis can range from mild diarrhea to fulminant colitis with toxic megacolon and sepsis (Alam & Madan, 2024). The pathogenic toxins A and B disrupt the colonic epithelial barrier, induce cytoskeletal disorganization and cell detachment, and trigger intense mucosal inflammation. Despite the use of targeted antibiotics and supportive care, recurrent and fulminant *C. difficile* colitis remains a major clinical challenge (Kordus, Thomas & Lacy, 2022; Zeng et al., 2022), making toxin-induced colitis an important experimental model for investigating acute epithelial injury and intestinal inflammation.

Intravenous immunoglobulin (IVIg) is a therapeutic preparation predominantly containing highly purified polyclonal IgG antibodies, derived from the pooled plasma of thousands of healthy donors (Dominguez-Andres et al., 2023; Gilardin, Bayry & Kaveri, 2015). Initially developed to treat primary immunodeficiency, IVIg is now recognized for its broad anti-inflammatory and immunomodulatory properties (Kobayashi et al., 2022; Lünemann, Nimmerjahn & Dalakas, 2015), and exerts its effects through multiple mechanisms, including neutralization of autoantibodies and pathogens, modulation of Fc receptors, suppression of inflammatory cytokines, and regulation of immune cells (Chaigne & Mouthon, 2017; Galeotti, Kaveri & Bayry, 2017; Zhang et al., 2023). These pleiotropic effects have expanded its clinical application to various diseases, including autoimmune and inflammatory disorders, malignancies, and secondary immunodeficiencies (Bayry et al., 2023; Burns & Franco, 2015; Lin, Chao & Chen, 2022).

Emerging studies suggest a potential therapeutic role of IVIg in intestinal inflammation, particularly in treatment-refractory cases or in patients contraindicated for immunosuppressive agents due to infection risk. However, the available evidence remains limited and heterogeneous, with variability in disease models, clinical scenarios, treatment protocols and mechanistic insights.

Therefore, this systematic review aims to comprehensively evaluate the effects of IVIg in animal and clinical studies of intestinal inflammation. We assess methodological quality, summarize therapeutic outcomes, and highlight knowledge gaps to inform future research directions and potential clinical applications of IVIg in intestinal inflammatory disorders.

## Methods

This systematic review was conducted in accordance with the Preferred Reporting Items for Systematic Reviews and Meta-analyses (PRISMA) 2020 guidelines (Page et al., 2021). The current protocol has been registered in the PROSPERO database with the registration number CRD420251051592.

### Search strategy

Two independent reviewers (TCC and XY) systematically searched PubMed, Web of Science, Embase and China National Knowledge Infrastructure (CNKI) for studies evaluating IVIg therapy in intestinal inflammation, including both animal and clinical studies. The search strategy was designed in consultation with a medical librarian to ensure methodological rigour and reproducibility. We used a combination of controlled vocabulary (MeSH terms in PubMed) and free-text keywords related to intravenous immunoglobulin and intestinal inflammatory conditions, including IBD, radiation- and chemotherapy-associated intestinal injury, and toxin-induced colitis.

The PubMed search strategy was as follows: ((“Immunoglobulins, Intravenous”[Mesh] OR IVIg[tiab] OR “intravenous immunoglobulin”[tiab])) AND (“Inflammatory Bowel Diseases”[Mesh] OR “Colitis”[Mesh] OR “Crohn Disease”[Mesh] OR “Radiation Injuries”[Mesh] OR colitis[tiab] OR enteritis[tiab] OR “inflammatory bowel disease*”[tiab] OR “ulcerative colitis”[tiab] OR “Crohn* disease”[tiab] OR intestinal[tiab] OR intestine[tiab] OR gastrointestinal[tiab] OR “radiation enteritis”[tiab] OR mucositis[tiab] OR toxin*[tiab]).

**Figure 1:**
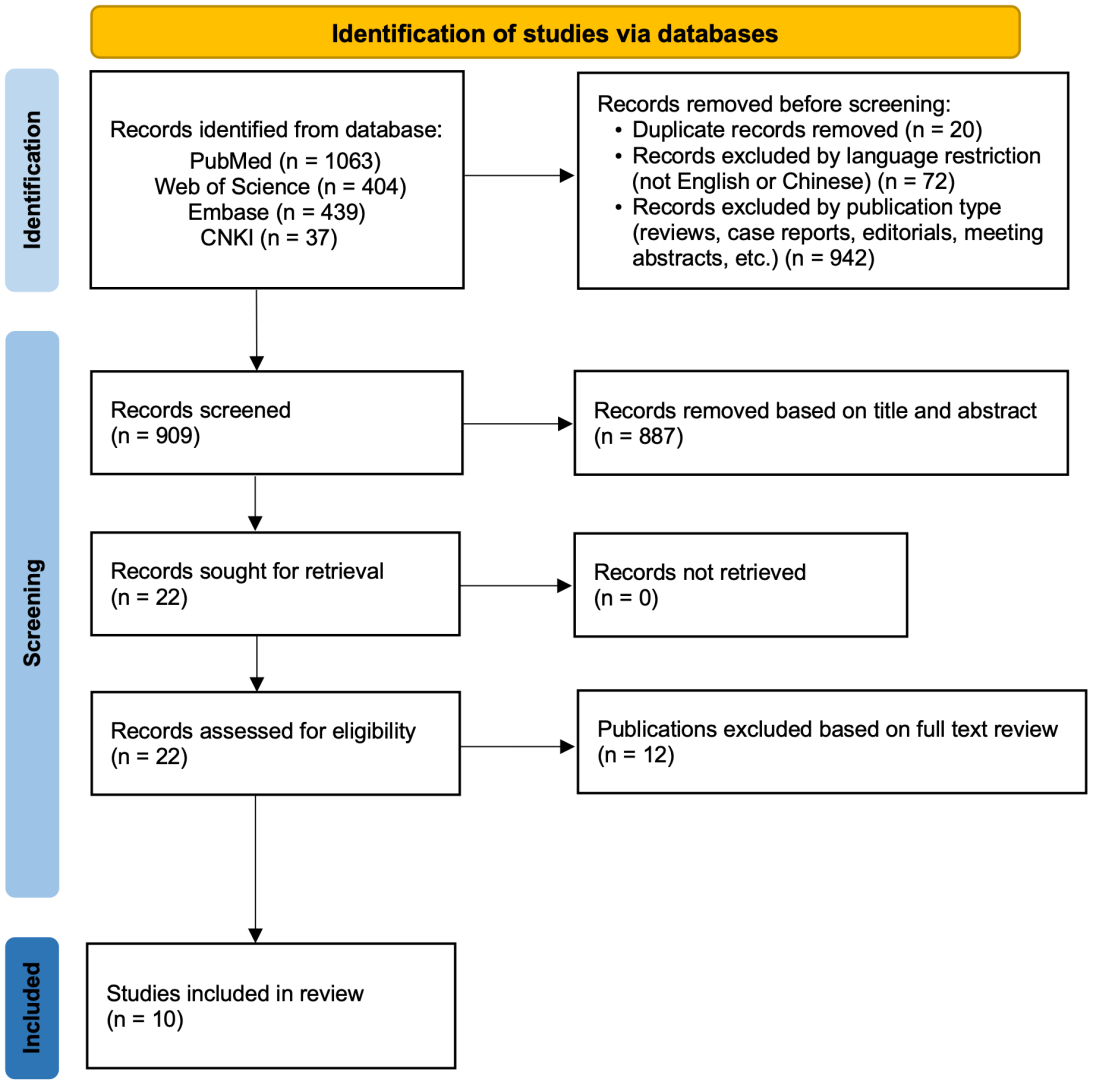
PRISMA Flow chart of selection of studies for the systematic review.

The primary search covered literature published from January 01, 2000 through April 17, 2025. An updated search was conducted on February 3, 2026 using the same databases and search strategy to ensure inclusion of the most recent eligible studies. The search syntax was adapted for Embase and Web of Science using the corresponding controlled vocabulary and database-specific operators. Duplicate records across various electronic databases were identified and removed using EndNote software (version 21). The methodology of study selection is illustrated in Fig. 1.

### Eligibility criteria

Studies were eligible for inclusion if they investigated the therapeutic effects of IVIg in either human patients diagnosed with IBD or animal models with experimentally induced intestinal inflammation. For animal studies, we did not restrict the underlying cause of intestinal injury and considered models in which intestinal inflammation was induced by immune-mediated mechanisms or by external insults, provided that intestinal inflammatory outcomes were reported. Only original research articles were included to ensure the analysis focused on the primary data and potential mechanisms.

Exclusion criteria were as follows: (1) Reviews, meta-analyses, case reports, editorials, and other secondary literature providing no primary evidence. (2) Conference abstracts lacking sufficient methodological detail. (3) Studies published in languages other than English or Chinese. (4) Studies without explicit descriptions of IVIg administration protocols. (5) Duplicates and studies with incomplete outcome reporting.

### Outcomes

Outcome measures in animal studies encompassed key parameters of intestinal pathology and immune responses, including: (i) histological assessments of mucosal integrity (*e.g.*, villus and crypt morphology, epithelial ulceration, goblet cell counts) (Charlet et al., 2019; He et al., 2024; Kozicky et al., 2019; Qin et al., 2023; Wang et al., 2023; Yan et al., 2025); (ii) tissue or serum levels of key pro- and/or anti-inflammatory cytokines (*e.g.*, TNF-α, IL-1β, IL-6, IL-10, IL-12/23p40) (Charlet et al., 2019; Kozicky et al., 2019; Qin et al., 2023; Yan et al., 2025); (iii) markers of oxidative stress and apoptosis (*e.g.*, glutathione peroxidase 4, 4-Hydroxynonenal, histone-associated DNA fragments) (He et al., 2024; Saito et al., 2011).

Outcome measures in clinical studies primarily focused on disease activity indices and clinical remission criteria, including the Harvey–Bradshaw Index (HBI) (Harvey & Bradshaw, 1980), Crohn’s Disease Activity Index (CDAI) (Best et al., 1976), partial Mayo score (Lewis et al., 2008; Schroeder, Tremaine & Ilstrup, 1987), and modified Pouchitis Disease Activity Index (mPDAI) (Sandborn et al., 1994; Shen et al., 2003). Definitions of clinical response and remission varied across studies but commonly involved threshold reductions in index scores, normalization of inflammatory markers, and the absence of hospitalizations, surgeries, or symptom exacerbation during follow-up.

### Data extraction

Two reviewers (TCC and XY) independently extracted data starting on May 14, 2025, using standardized data extraction forms. Any discrepancies between reviewers were resolved by discussion. If consensus could not be reached, a third reviewer (ZKW) served as the adjudicator to make the final decision. Following the updated literature search on February 3, 2026, which identified no new eligible studies for inclusion, the extracted dataset was confirmed to remain current and unchanged. For animal studies, information on publication data, model characteristics, interventions, research methods, outcome measures and mechanistic insights (*e.g.*, cytokine modulation, receptor pathways) was extracted. For clinical studies, extracted data included publication data, study design, sample size, participant characteristics, interventions, main outcome measures, and limitations.

### Risk of bias assessment

The risk of bias in animal studies was evaluated using the SYstematic Review Centre for Laboratory animal Experimentation (SYRCLE) Risk of Bias tool (Hooijmans et al., 2014). For human observational studies (retrospective cohort and case series), the Risk Of Bias In Non-randomized Studies of Interventions (ROBINS-I) tool was applied to assess methodological limitations (Sterne et al., 2016). Two independent reviewers (TCC and XY) conducted all assessments, with discrepancies resolved through discussion or consultation with a third reviewer (ZKW).

## Results

A total of ten studies met the inclusion criteria for this systematic review, including seven animal studies and three clinical studies (Charlet et al., 2019; Chrissafidou, Malek & Musch, 2007; He et al., 2024; Horton et al., 2017; Kozicky et al., 2019; Merkley et al., 2015; Qin et al., 2023; Saito et al., 2011; Wang et al., 2023; Yan et al., 2025).

### Animal studies

The seven selected studies, published between 2011 and 2025, employed mouse models of intestinal inflammation, predominantly using C57BL/6 (including the C57BL/6J substrain) and BALB/c mice aged 6–12 weeks, with both sexes represented. These studies assessed the potential therapeutic effects of IVIg in various experimental models of intestinal inflammation, such as dextran sulfate sodium (DSS)-induced colitis, radiation-induced enteritis, chemotherapy-induced mucositis (*via* doxorubicin or oxaliplatin), and *C. difficile* toxin-induced intestinal injury. Study characteristics and risk of bias assessment are summarized in Tables 1 and 2, respectively.

**Table 1:** Characteristics of the animal studies.

Source	Country	Study design	Species	Model	Weight	Interventions	Research methods	Outcome measures	Potential mechanism
Charlet et al. (2019)	France	Controlled trial	C57BL/6 mice (Female, 8–10 weeks old)	DSS-induced colitis (fungal challenge with *C. albicans*)	NR	IVIg (800 mg/kg, i.p.), daily for 7 days	MALDI-TOF MS, Histological analysis, ELISA, Spectrophotometry	- ↓Inflammation - ↓*E. coli* and *E. faecalis*, ↓*C. albicans*	↓IL-6, ↑ IL-10, ↑ PPARγ expression in innate immune cells
Kozicky et al. (2019)	Canada	Controlled trial	C57BL/6 mice (M&F, 8–12 weeks old)	DSS-induced colitis	NR	IVIg (1 g/kg, i.p.), every other day for 4 doses, starting day 0	HE staining, ELISA, RNAscope	- Reduced histological damage - ↑ IL-10, ↓ IL-12, ↓ IL-23 p40, ↓ IL-1*β*	Effect dependent on macrophage-derived IL-10 production
He et al. (2024)	China	Controlled trial	C57BL/6J mice (Male, 6–8 weeks old)	Radiation-induced enteritis	22–25 g	IVIg (500 mg/kg, i.v.) within 1 h after radiation	IHC staining, Western blotting, Proteomics analysis, TEM	- Preserved villi - ↑ ZO-1 and occludin - ↓ lipid and iron accumulation - ↑ GPX4 ↓ 4HNE	Activate the mTOR pathway to reduce ferroptosis and promote epithelial junctions’ formation
Wang et al. (2023)	China	Controlled trial	C57BL/6J mice (M&F, 6-8 weeks old)	Radiation-induced enteritis	NR	IVIg (375 mg/kg, i.v.) every 3 days ×5 doses	Bacterial configuration analysis, Metabolomics analysis, HE&PAS staining, ELASA	- ↑ Villi height, goblet cells (females) - IVIg + *Lachnospiraceae*/ hypoxanthine effective in males	FcγR-mediated phagocytosis *via Lachnospiraceae*/ hypoxanthine axis
Qin et al. (2023)	China	Controlled trial	BALB/c, (Male, 6–8 weeks old)	Oxaliplatin-induced intestinal injury	22–24 g	IVIg (800 mg/kg, i.p.) for 7 days	HE&PAS staining, Luminex assay	- Reduced histological damage - ↑ goblet cells - ↓ TNF-α, IFN-γ ↑ IL-10, IL-13	Modulation of inflammatory cytokines and mucosal repair
Yan et al. (2025)	China	Controlled trial	C57BL/6J, (Male, 7–8 weeks old)	Doxorubicin-induced mucositis	20–24 g	IVIg (500 mg/kg, i.v.) within 2 h and 3 days after Dox intervention	IHC and IF staining, Proteomics analysis, TEM, Western blotting	- Preserved crypt/villi structure - Retore TJ proteins - ↓TNF-α, IL-6, IFN-γ, IL-27, IL-2, Chitinase3-like 1 and TNF RII	Inhibition the Syk/PI3K/Akt axis to protect intestinal barrier integrity and restrain ferroptosis
Saito et al. (2011)	Japan	Controlled trial	BALB/c, (Female, 6 weeks old)	*Clostridioides difficile* toxin-induced enteritis	NR	IVIg (100 mg/kg/day, i.p.), given at −12 h, 0 h, +6 h, or +12 h relative to toxin administration	Evans blue dye extravasation assay, ELISA	- ↓ Intestinal vascular permeability - ↓ Apoptosis marker (histone-associated DNA fragments)	Not experimentally confirmed

**Notes.**

DSS dextran sulfate sodium C. albicans *Candida albicans* NR not reported i.p. intraperitoneal injection; MALDI-TOF MS matrix-assisted laser desorption/ionization time-of- flight mass spectrometry ELISA enzyme-linked immunosorbent assay E. coli *Escherichia coli* E. faecalis *Enterococcus faecalis* IL interleukin PPARγ peroxisome proliferator-activated receptor gamma M&F male and female HE hematoxylin and eosin i.v. intravenous injection IHC immunohistochemistry TEM transmission electron microscopy ZO-1 zonula occludens-1 GPX4 glutathione peroxidase 4 4HNE 4-hydroxynonenal mTOR mechanistic target of rapamycin PAS periodic acid-Schiff TNF tumour necrosis factor IFN interferon FcγR Fc gamma receptor Dox doxorubicin IF immunofluorescence TJ tight junction TNF-RII tumour necrosis factor receptor II Syk spleen tyrosine kinase PI3K phosphoinositide 3-kinase Akt protein kinase B

**Table 2:** Quality assessment of animal studies (SYRCLE tool).

	Sequence generation (selection)	Baseline characteristics (selection)	Allocation concealment (selection)	Random housing (performance)	Blinding (Performance)	Random outcome assessment (detection)	Blinding (detection)	Incomplete outcome data (attrition)	Selective outcome reporting (reporting)	Other biases
Charlet et al. (2019)	U	L	U	U	U	U	U	L	L	L
Kozicky et al. (2019)	L	L	U	U	H	U	L	L	L	L
He et al. (2024)	U	L	U	U	U	U	U	L	L	L
Wang et al. (2023)	L	L	U	U	U	U	U	L	L	L
Qin et al. (2023)	L	L	U	U	U	U	U	L	L	L
Yan et al. (2025)	L	L	U	U	U	U	L	L	L	L
Saito et al. (2011)	U	L	U	U	U	U	U	L	L	L

**Notes.**

L low risk H high risk U unclear risk

#### DSS-induced colitis models

Two studies explored the effects of IVIg in DSS-induced colitis models (Charlet et al., 2019; Kozicky et al., 2019). Across these experiments, IVIg was administered at relatively high doses *via* the intraperitoneal route in the early phase of colitis induction. In both models, IVIg administration attenuated DSS-induced colitis, with less body weight loss, reductions in clinical symptoms and improved histological appearance of colonic mucosa compared with DSS alone.

In a *Candida albicans* (*C. albicans*)-colonized DSS model (Charlet et al., 2019), IVIg suppressed the overgrowth of aerobic bacteria such as *Escherichia coli* (*E. coli*) and *Enterococcus faecalis* (*E. faecalis*), and facilitated clearance of *C. albicans*. The beneficial effects were associated with decreased interleukin (IL)-6 and increased IL-10 levels, as well as upregulation of peroxisome proliferator-activated receptor gamma (PPARγ). In the other DSS model, IVIg or IVIg-treated macrophages decreased intestinal inflammation and collagen accumulation, while shifting cytokine production towards an anti-inflammatory profile; protection was lost when macrophage IL-10 production was genetically ablated (Kozicky et al., 2019). Collectively, these findings suggest IVIg confers anti-inflammatory effects in DSS-induced colitis models through innate immune modulation.

#### Radiation-induced enteritis models

Two studies evaluated the protective effects of IVIg in murine models of radiation-induced enteritis. He et al. (2024) exposed male mice to abdominal X-ray irradiation (12–14 Gy) and showed that IVIg administration significantly preserved intestinal epithelial integrity, as evidenced by increased villus height and restored expression of tight junction proteins. Furthermore, IVIg inhibited ferroptosis in intestinal epithelial cells, and these protective effects were dependent on activation of the mechanistic target of rapamycin (mTOR) pathway, as co-treatment with rapamycin reversed the benefits. Complementary *in vitro* experiments using irradiated intestinal organoids further supported these findings.

Wang et al. (2023) investigated sex-dependent effects of IVIg in irradiation-induced intestinal and hematopoietic injury in mice of both sexes. IVIg treatment improved survival, reduced intestinal inflammation, and enhanced intestinal barrier function, particularly in female mice. In male mice, comparable protective effects were achieved only when IVIg was combined with either *Lachnospiraceae* or hypoxanthine supplementation. Mechanistic analysis revealed that these effects of IVIg were attributed to the activation of Fcγ receptor-mediated phagocytosis, driven by the *Lachnospiraceae*/hypoxanthine/phospholipase D1 (PLD1) axis. Together, these findings suggest that IVIg mitigates radiation-induced intestinal injury through both direct epithelial protection and microbiota-mediated immune modulation.

#### Chemotherapy-induced intestinal injury

Two studies (Qin et al., 2023; Yan et al., 2025) assessed the protective effects of IVIg in mouse models of chemotherapy-induced intestinal injury, using oxaliplatin or doxorubicin. IVIg treatment effectively preserved intestinal morphology, including villus and crypt structure, and improved goblet cell numbers. It also restored tight junction proteins and reduced markers of epithelial cell ferroptosis and inflammation. In the oxaliplatin-induced model, IVIg facilitated mucosal repair *via* an anti-inflammatory shift, whereas in the doxorubicin-induced model, protection was mediated through inhibition of the spleen tyrosine kinase (Syk)/phosphoinositide 3-kinase (PI3K)/protein kinase B (Akt) signaling pathway. These findings suggest that IVIg may alleviate chemotherapy-induced mucosal injury through both immune modulation and suppression of epithelial cell death.

#### Toxin-induced intestinal damage

In a murine model of *C. difficile* toxin-induced enteritis, a form of infectious colitis (Saito et al., 2011), C57BL/6 mice were injected intraperitoneally with a filtered preparation containing *C. difficile* toxins, resulting in acute intestinal epithelial damage, increased vascular permeability, and high mortality. Although clinically this represents infectious colitis, in the present review we classify it within the toxin-induced intestinal injury category because epithelial damage is primarily driven by toxin A and B. IVIg administration at different time points relative to toxin exposure conferred significant protection, with maximal protection when IVIg was given concurrently with the toxin. IVIg treatment improved survival, reduced intestinal vascular leakage, and decreased epithelial apoptosis as measured by histone-associated DNA fragmentation. While the Fas/FasL pathway was implicated in the pathogenesis of toxin-induced injury, the precise mechanism by which IVIg exerted its protective effect was not experimentally confirmed in this study.

### Human studies

**Figure 2:**
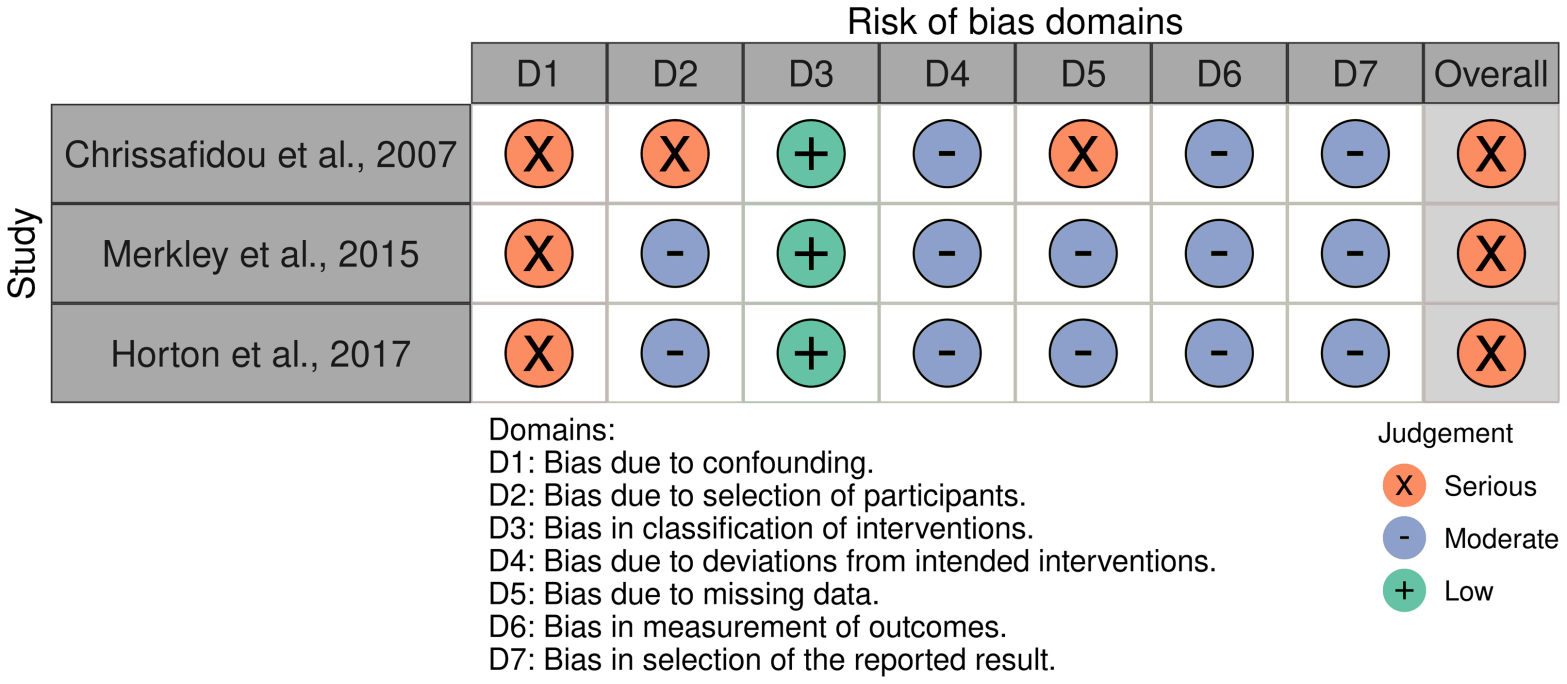
Risk of bias assessment of included clinical studies using the ROBINS-I tool.

The detailed study characteristics of the included human studies are presented in Table 3, with the risk of bias assessment summarized in Fig. 2. All three were retrospective observational studies conducted between 2007 and 2017 in small cohorts of patients with difficult-to-treat disease, including steroid-resistant Crohn’s disease, refractory IBD (Crohn’s disease, ulcerative colitis and pouchitis) and IBD with contraindications to standard immunosuppression (Chrissafidou, Malek & Musch, 2007; Horton et al., 2017; Merkley et al., 2015). In all three studies, IVIg was used as adjunctive or rescue therapy on top of existing treatments rather than as first-line induction.

**Table 3:** Characteristics of the human studies.

Source	Country	Study design	Trial size	Type	Mean age	Interventions	Main outcome measures	Results	Limitations
Chrissafidou, Malek & Musch (2007)	Germany	RCS	19	Steroid-resistant CD patients (CDAI >150, mean steroid dose 18.2 mg/day)	44 ± 16 years	IVIg (10 g/day ×7 days or 72 or 90 g over 8 or 10 days) + 5-ASA/SASP + corticosteroids	CDAI (weekly assessments, from 3 weeks pre-therapy to 4 weeks post)	73.7% achieved remission (CDAI drop > 100); sustained remission (mean 20.6 months) in 8 followed patients; no major adverse events	Small sample size, retrospective data
Merkley et al. (2015)	America	RCR	24	Refractory IBD (23 CD, 1 UC) who failed or had contraindications to standard therapy, or active infection	Female: 41 years; Male: 39 years	IVIg (0.4 g/kg, i.v.) ×3 days; monthly maintenance; biweekly if suboptimal response	Clinical response: HBI ≥ 3 or CRP improvement > 25%; Clinical remission: HBI <5, no hospitalizations or surgeries after administration, or symptom alleviation	IVIg induced response in 16 patients (67%), with 3 (12.5%) achieving remission; CRP significantly decreased from 19 mg/dL to 7.5 (*p* < 0.05), and HBI improved from 8 to 6 (*p*= NS)	Small cohort, lack of randomized controlled trials
Horton et al. (2017)	America	RCS	54	Refractory IBD patients (23 CD, 15 UC, 16 pouchitis)	42 ± 16 years	IVIg (0.4 g/kg, i.v.): 1 × (*n* = 37), 2 × (*n* = 11), ≥3 × (*n* = 6)	UC: partial Mayo Score; CD: HBI; Pouchitis: mPDAI	All disease scores improved for all patients (HBI, *p* = 0.07; partial Mayo score, *p* = 0.02; mPDAI, *p* = 0.08)	Non-standardized IVIg dosing, tertiary-center selection bias, uncontrolled design

**Notes.**

RCS retrospective case series CD Crohn’s disease CDAI Crohn’s disease activity index 5-ASA/SASP 5-aminosalicylic acid and sulfasalazine RCR retrospective chart review IBD inflammatory bowel disease UC ulcerative colitis i.v. intravenous injection HBI Harvey–Bradshaw index CRP C-reactive protein NS not significant mPDAI modified pouchitis disease activity index

Across these cohorts (sample sizes ranging from fewer than 20 to around 50 patients), a proportion of patients experienced short-term clinical improvement or remission according to CDAI (Chrissafidou, Malek & Musch, 2007), HBI (Horton et al., 2017; Merkley et al., 2015), partial Mayo score (Horton et al., 2017) or mPDAI (Horton et al., 2017), and some reports also described reductions in inflammatory markers such as C-reactive protein (CRP) and endoscopic improvement after IVIg (Merkley et al., 2015). However, responses were inconsistent and not universally durable; in the largest series, about half of the patients eventually required bowel surgery despite initial improvement, and *C. difficile* infection emerged as a predictor of IVIg treatment failure (Horton et al., 2017). All three studies were uncontrolled, involved small and heterogeneous patient populations, and used non-uniform IVIg dosing regimens and outcome definitions, which substantially limit the strength and generalizability of any conclusions regarding clinical efficacy.

## Discussion

This systematic review synthesized evidence from seven animal studies and three clinical studies evaluating IVIg in models and clinical settings of intestinal inflammation. Across various animal models, IVIg consistently demonstrated anti-inflammatory and epithelial-protective properties. Key outcomes included attenuation of histopathological damage, reduction of pro-inflammatory cytokines and restoration of tight junction integrity. By contrast, the clinical data were limited to three small, retrospective and uncontrolled cohorts of highly selected patients with steroid-resistant or medically refractory IBD, or with contraindications to standard immunosuppression. Although a proportion of patients experienced short-term improvements in disease activity scores, inflammatory markers or endoscopic findings, responses were inconsistent and not durable in all cases. Taken together, these findings suggest that IVIg has the potential to modulate intestinal inflammation in preclinical models, while the current human data remain preliminary and do not allow firm conclusions regarding clinical efficacy.

Although both animal and clinical data suggest that IVIg can modulate intestinal inflammation, the strength and nature of the evidence differ substantially. Animal studies were conducted in tightly controlled experimental settings, often with defined timing of IVIg administration relative to injury, restricted comorbidities and standardized readouts of histological damage, barrier integrity and inflammatory mediators. These models allowed the identification of potential mechanistic pathways and consistently showed attenuation of acute intestinal injury. By contrast, the clinical studies were retrospective, uncontrolled and methodologically heterogeneous, enrolling small numbers of patients with chronic, relapsing-remitting and highly refractory IBD, frequently after multiple prior therapies. In this setting, IVIg was used as adjunctive or rescue treatment, and clinical responses were variable and not always durable. These differences in study design, patient populations and timing of intervention underscore a substantial translational gap between experimental models and real-world clinical practice and highlight the need for rigorously designed prospective, ideally controlled, clinical studies to evaluate the role of IVIg in human intestinal disease.

At the clinical level, however, the efficacy of IVIg remains uncertain. Across the three cohorts, only a minority of patients achieved sustained clinical remission, many required dose escalation or additional therapies over time (Merkley et al., 2015), and in the largest series a substantial proportion ultimately progressed to bowel surgery (Horton et al., 2017). Moreover, some disease activity indices did not improve significantly, and concomitant *C. difficile* infection emerged as a predictor of IVIg treatment failure (Horton et al., 2017). Overall, these observations suggest that IVIg may provide short-term symptomatic or stabilizing benefit in selected refractory cases, but the available data are insufficient to demonstrate a durable disease-modifying effect or to support its routine use in clinical practice.

At the mechanistic level, the animal studies included in this review suggest that IVIg acts at multiple points along the immune-epithelial-microbial axis in the gut. In DSS-induced colitis, protection depended on macrophage-derived IL-10, as IVIg or IVIg-treated macrophages ameliorated inflammation, whereas the benefit was lost when IL-10 signaling was disrupted (Charlet et al., 2019; Kozicky et al., 2019). In radiation- and chemotherapy-induced intestinal injury, several studies linked the protective effects of IVIg to modulation of regulated cell death pathways in intestinal epithelial cells, including ferroptosis *via* mTOR-GPX4 signaling and inhibition of Syk/PI3K/Akt-mediated injury (He et al., 2024; Qin et al., 2023; Yan et al., 2025). Beyond direct epithelial effects, IVIg also interacted with intestinal microbiota and innate immune system: in a radiation model, IVIg-mediated protection was associated with Fcγ receptor-dependent phagocytic activity driven by a *Lachnospiraceae*/hypoxanthine/PLD1 axis, with clear sex-specific differences (Wang et al., 2023). In the *C. difficile* toxin model, IVIg reduced vascular leakage and epithelial apoptosis (Saito et al., 2011). Taken together, these findings support the concept that IVIg can dampen injurious innate immune responses, protect epithelial integrity and modulate host-microbiota interactions in experimental intestinal inflammation. However, these mechanistic insights are derived almost entirely from preclinical models, with very limited validation in patients, and should therefore be regarded as hypothesis-generating rather than established mechanisms in human IBD.

An important discrepancy emerges when comparing the *C. difficile* toxin-induced murine model (Saito et al., 2011) with the clinical data from Horton et al. (2017). In the experimental setting, IVIg administered around the time of toxin exposure improved survival and reduced intestinal vascular leakage and epithelial apoptosis in otherwise healthy mice challenged with *C. difficile* toxin-containing preparations. By contrast, in the largest clinical cohort of refractory IBD inpatients treated with IVIg, concomitant *C. difficile* infection was associated with a higher risk of subsequent bowel resection despite initial improvement in disease activity indices. This contrast likely reflects fundamental differences between an acute toxin-challenge model without antibiotics or chronic inflammation and the complex clinical scenario of hospitalized patients with longstanding, treatment-refractory IBD, active infection, multiple comorbidities and concurrent antimicrobial and immunosuppressive therapies. These considerations reinforce that current management of *C. difficile* infection should continue to rely on guideline-directed antibiotic and microbiota-based strategies, and that IVIg cannot be recommended as a replacement for standard care. At most, IVIg may be considered as an adjunctive or rescue option in highly selected, refractory cases, but the available evidence to support this approach remains very limited.

From a clinical perspective, it is also important to situate IVIg within the current therapeutic landscape of intestinal inflammation, particularly IBD. Over the past decade, treatment options have expanded to include multiple biologic agents targeting TNF-α, integrins and IL-12/23p40, IL-23p19 (Verstockt et al., 2023), as well as small-molecule therapies such as Janus kinase (JAK) inhibitors and sphingosine-1-phosphate (S1P) receptor modulators (Noor, Bourke & Subramanian, 2024; Scott et al., 2016), many of which are supported by large randomized controlled trials and real-world data and are now incorporated into international guidelines as standard options for moderate-to-severe disease (Lamb et al., 2019; Louis et al., 2024; Sandborn et al., 2013; Sandborn et al., 2012; Torres et al., 2020). Emerging strategies such as dual targeted therapy and biomarker-guided, personalized treatment algorithms are further refining care for patients with complex IBD (Altieri et al., 2025; Graham & Xavier, 2020). By contrast, IVIg has only been used off-label in small, retrospective and uncontrolled cohorts as adjunctive or rescue therapy in highly refractory or contraindicated cases. In this context, IVIg should not be viewed as a competitor to established advanced therapies but, at most, as a potential niche option when guideline-recommended biologics and small molecules are ineffective, contraindicated or unavailable, a role that still requires formal evaluation.

A further limitation of the available evidence relates to the risk of bias in both the animal and clinical studies. In the preclinical literature, SYRCLE-based assessment showed that several key domains were predominantly rated as “unclear” risk, including sequence generation, allocation concealment, random housing, blinding of caregivers and investigators, and random outcome assessment (Table 2). Only one study reported a high risk of performance bias, and most did not clearly describe procedures to minimize selection or detection bias. By contrast, incomplete outcome data, selective outcome reporting and other biases were generally judged to be at low risk. Overall, however, the lack of transparent reporting on randomization and blinding means that treatment effects in these animal experiments may be overestimated, and their reproducibility remains uncertain. Similarly, the three human studies were rated as having serious to critical risk of bias on ROBINS-I, mainly due to confounding by indication, lack of control groups, retrospective design, incomplete reporting of co-interventions and outcomes, and potential selection bias. As a result, causal inferences about the clinical efficacy of IVIg cannot be drawn, and any apparent improvements must be interpreted with great caution, acknowledging that effect estimates may be inflated and that alternative explanations, including changes in background therapy or regression to the mean, cannot be excluded.

This systematic review has several strengths. We followed PRISMA guidance, searched multiple databases in two languages, and applied standardized tools (SYRCLE and ROBINS-I) to appraise the risk of bias in both animal and clinical studies, allowing a structured comparison across distinct models of intestinal injury and human IBD cohorts. Nonetheless, important limitations must be acknowledged. The number of eligible studies was small, with substantial heterogeneity in animal models, IVIg dosing regimens, timing of administration and outcome measures, precluding meta-analysis and limiting quantitative synthesis. All three clinical studies were retrospective, uncontrolled and at serious to critical risk of bias, and the exclusion of case reports, while avoiding anecdotal over-interpretation, may have omitted rare but potentially informative experiences in highly selected patients. In addition, the inclusion of mechanistically distinct conditions (IBD, radiation enteritis, chemotherapy-induced mucositis and toxin-mediated injury) introduces clinical heterogeneity that restricts the generalizability of any overarching conclusions. In light of these considerations, the present review should be regarded primarily as hypothesis-generating. Preclinical data support the concept that IVIg can modulate intestinal inflammation and epithelial injury, but current human evidence is too limited and uncertain to justify routine clinical use. Future work should focus on improving the internal validity and reporting quality of animal studies and, critically, on rigorously designed prospective, preferably controlled, clinical trials in clearly defined patient populations (for example, steroid-refractory IBD with contraindications to standard immunosuppression), ideally incorporating mechanistic endpoints and biomarkers to better identify those who might derive meaningful benefit from IVIg.

## Conclusions

This systematic review summarizes the currently available animal and clinical evidence on the use of IVIg in intestinal inflammation. In preclinical models, IVIg has been shown to ameliorate disease severity and epithelial injury through several proposed mechanisms, including IL-10–dependent modulation of macrophages and effects on innate immune and cell death pathways, although these findings arise from heterogeneous studies and therefore need to be interpreted with caution. Human evidence is limited to three small, retrospective, uncontrolled cohorts in highly selected, refractory IBD populations, in which some patients experienced clinical improvement, but responses were variable and subject to potential confounding. Taken together, the present data suggest that IVIg cannot yet be regarded as a standard treatment option for IBD or other intestinal inflammatory conditions and that any use in clinical practice is best reserved for carefully selected, refractory situations in which guideline-recommended therapies are ineffective or contraindicated. If IVIg is to be further explored in this context, future work is likely to be most informative if it focuses on improving the rigour and transparency of preclinical studies and on small, well-designed prospective clinical studies in clearly defined niche indications, which may help to clarify whether IVIg has a limited but meaningful role within the expanding therapeutic armamentarium for intestinal inflammation.

##  Supplemental Information

10.7717/peerj.21024/supp-1 Supplemental Information 1 PRISMA checklist

## Abbreviations

IVIg: Intravenous immunoglobulin
IBD: Inflammatory bowel disease
UC: Ulcerative colitis
CD: Crohn’s disease
DSS: Dextran sulfate sodium
TNF: Tumour necrosis factor
HBI: Harvey–Bradshaw index
CDAI: Crohn’s Disease Activity Index
mPDAI: Modified Pouchitis Disease Activity Index
CRP: C-reactive protein
IL: Interleukin
PPARγ: Peroxisome proliferator-activated receptor gamma
mTOR: Mechanistic target of rapamycin
PLD1: Phospholipase D1
Syk: Spleen tyrosine kinase
PI3K: Phosphoinositide 3-kinase
Akt: Protein kinase B
GPX4: Glutathione peroxidase 4
JAK: Janus kinase
S1P: Sphingosine-1-phosphate

## Additional Information and Declarations

### Data Availability

The following information was supplied regarding data availability: This is a systematic review.
